# Psychosocial Factors Associated with Longevity in the United States: Age Differences between the Old and Oldest-Old in the Health and Retirement Study

**DOI:** 10.4061/2011/530534

**Published:** 2011-10-17

**Authors:** Jennifer A. Ailshire, Eileen M. Crimmins

**Affiliations:** Davis School of Gerontology, University of Southern California, 3715 McClintock Avenue, Room 218C, Los Angeles, CA 90089-0191, USA

## Abstract

Recent growth in the number of adults surviving to advanced ages raises questions about the quality of life associated with increased longevity. Psychosocial factors have received relatively little attention in research on quality of life among the oldest-old. This study uses nationally representative data on older US adults to examine how social relationships, feelings of loneliness, and satisfaction with life and the aging experience differ between the oldest-old, those who have survived to age 90 or older, and older adults in their 70s. We find that the oldest-old are able to maintain social relationships with family and friends and receive more social support than younger elderly adults. Yet, the oldest-old are more likely to feel lonely due to their greater rates of widowhood. Satisfaction with life was higher among the oldest-old, but the oldest-old had more negative perceptions of the aging experience. Psychosocial dimensions of longevity should be considered in research on quality of life among the oldest-old.

## 1. Introduction

As a result of recent demographic changes, such as declining mortality rates among older adults, reaching advanced old age has become an increasingly common experience in the US and around the world [1–3]. The rapid growth in the number of very old people raises questions about the quality of additional years of life lived by those achieving exceptional longevity. The World Health Organization has declared that “increased longevity without quality of life is an empty prize” [4]. Although much of the research thus far on longevity and quality of life has emphasized health and functioning, we argue that a more comprehensive understanding of quality of life at advanced old ages could be achieved by additionally considering psychosocial well-being.

Traditional approaches to studying quality of life among those who reach advanced old age are rooted in a biomedical paradigm that emphasizes the avoidance of disease and cognitive and physical declines [5]. There is concern, for instance, that those who survive to very old age spend their remaining years in a state of poor health and functioning and, therefore, have a poor quality of life [6, 7]. Prior research on long-lived individuals indeed confirms that among individuals who survive to exceptional old age there is a high prevalence of disease and disability as well as impaired cognitive performance [8–12]. 

However, aging is a multidimensional concept, and psychosocial factors that assess psychological and social well-being should be included in conceptual frameworks used to understand the aging process [13]. It is possible, for instance, that those experiencing health and functioning declines that accompany the aging process are still able to maintain a high quality of life with respect to social and psychological well-being. Thus, while the predominant conceptual framework for understanding the aging process places a strong emphasis on health and functioning, a more inclusive conceptualization of quality of life in advanced ages should also focus on psychosocial well-being. 

Gerontologists have been interested in the psychosocial dimensions of quality of life in older adults for some time. In his conceptualization of the “good life”, Lawton argued that psychosocial indicators of well-being are inextricably linked with health and functioning in determining quality of life among older adults [14]. Psychosocial dimensions of well-being, such as social relationships, feelings of loneliness, and satisfaction with life, are important factors to consider in the measurement of quality of life among older adults [14, 15]. Prior research has established that social relationships, feelings of loneliness, and a sense of life satisfaction are not only predictive of longevity [16–18], but are also important for determining health-related quality of life among older adults [19–21]. Social connectedness and feeling positive about one's life may be particularly important for quality of life in very old age when health-related quality of life has declined. Yet, there has been very little empirical research on the psychosocial factors associated with longevity. 

Because there are few sources of data on the oldest-old, and even less research on the psychosocial characteristics of the longest lived, our understanding of longevity and quality of life is limited. A few studies have compared the psychosocial well-being of US centenarians to younger elderly adults, finding that centenarians have fewer social relationships and less frequent social contact than their younger elderly counterparts [22] and that loneliness is more prevalent in extreme old age [23]. These studies of centenarians and older adults living in the state of Georgia indicate that long-lived individuals living in this southeastern state in the US have comparatively worse quality of life than younger old adults. In contrast, a study comparing the psychosocial well-being of Italian centenarians to younger elderly Italians found that centenarians reported having more social support and greater life satisfaction [24]. 

Studies of social and psychological factors among older US adults indicate that quality of life is worse among the oldest-old compared to the younger elderly. Age-related differences in social characteristics and health status may explain why the oldest-old have worse quality of life. For instance, as the oldest-old outlive family and friends they experience a contraction in their network of social relationships and, consequently, may have fewer social contacts and feel more socially isolated than younger adults. Furthermore, higher rates of disease and disability among the oldest-old [8–12] may impede their ability to live independent, socially engaged lives, which could result in fewer social interactions, greater feelings of loneliness, and less satisfaction with their lives. 

The present study uses a nationally representative sample of older US adults to examine age differences in psychosocial characteristics between the old, those in their 70s, and oldest-old, those aged 90 or older. This study has two aims. The first is to determine whether social relationships, feelings of loneliness, satisfaction with life, and perceptions of the aging experience differ for those who have achieved exceptional longevity, compared with younger elderly adults. Based on findings from prior research on psychosocial factors associated with longevity, we expect that the oldest-old adults will be less socially connected but will report more social support than their younger counterparts. In addition, the oldest-old will express greater life satisfaction compared to younger elderly adults, though we expect the oldest-old will be less satisfied with the aging process. The second aim is to identify social and health characteristics that contribute to age differences in social and psychological well-being. We expect that age differences in feelings of loneliness, satisfaction with life, and perceptions of the aging experience are largely the result of age-related changes in social factors and health status.

## 2. Methods

### 2.1. Data

This study uses data from the Health and Retirement Study (HRS), a nationally representative ongoing survey of US adults over the age of 50. The HRS is designed to monitor changes in physical, functional, and cognitive health associated with aging. In 2006, the HRS also began collecting information on psychosocial characteristics of older adults. A random one-half of HRS households were selected to complete a self-administered psychosocial questionnaire in 2006, with the other half of the sample selected for participation in 2008.

The psychosocial questionnaire was administered to 8,568 respondents in 2006 and 7,500 respondents in 2008 who were living in the community. Because the primary aim of this study is to characterize the psychosocial factors of longevity, we focus on the oldest-old respondents who were between 90 and 104 years of age at the time of the interview. Survival to age 90 and beyond is relatively uncommon in older cohorts. Among those born in 1900, for instance, only about 5% of men and 14% of women survived to age 90 according to cohort life tables [1]. As a comparison, we also examine psychosocial characteristics of respondents who have achieved average survival and were age 70–79 at the time of the interview. The analytic sample comprised 4,187 older adults, aged 70–79, and 281 oldest-old adults, aged 90–104, who completed the psychosocial questionnaire.

### 2.2. Measures

Sociodemographic measures used to characterize the sample include gender, race/ethnicity, educational attainment, marital status, and living arrangements. 

Health status is assessed with the number of comorbidities and the number of limitations in activities of daily living (ADLs). Number of comorbidities counts the number of doctor diagnosed diseases and chronic conditions reported by respondents or their proxies, including (1) high blood pressure or hypertension, (2) diabetes or high blood sugar, (3) cancer or a malignant tumor of any kind except skin cancer, (4) chronic lung disease such as chronic bronchitis or emphysema (excluding asthma), (5) heart attack, coronary heart disease, angina, congestive heart failure, or other heart problem, and (6) stroke. ADL limitations were assessed with a count of the number of six major life activities the respondent had difficulty performing, including walking across a room, dressing, bathing, eating, getting in and out of bed, and using the toilet. 

We assess social relationships and quality of life among older and oldest-old adults with measures that reflect the extent and quality of respondents' relationships with others, their feelings of loneliness, and their satisfaction with life in general and the aging experience in particular.

#### 2.2.1. Social Relationships

We examine measures that capture the extent of social relationships, including frequency of contact with family and friends, the number of close relationships, and levels of social support and strain.

Frequency of social contact includes contact with nonhousehold children, nonhousehold family members, and friends. Questions about these forms of social contact were only asked in the 2008 survey. We created separate scales of frequency of contact with children, family, and friends. The scales were created by averaging responses to the following two questions: “On average, how often do you meet up with any of your children/family members/friends, not counting any who live with you?” and “On average, how often do you speak on the phone with any of your children/family members/friends, not counting any who live with you?” The response options were less than once a year or never = 1, once or twice a year = 2, every few months = 3, once or twice a month = 4, once or twice a week = 5, and three or more times a week = 6. Scale scores ranged from 1 to 6, with higher scores indicating more contact. The alpha coefficient of reliability for each of the scales was  .66 for contact with children,  .75 for contact with family, and  .65 for contact with friends. 

We also included separate measures for the number of close relationships with children, family members, and friends. Respondents were asked about their children, family members, and friends, “How many…would you say you have a close relationship with?” We coded responses so the number of close relationships ranged from 0 to 10 or more. 

We assessed the quality of social relationships with measures of social support and relationship strain from spouses/partners, children, other family members, and friends. Social support was measured with the following three items: (1) How much do they really understand the way you feel about things? (2) How much can you rely on them if you have a serious problem? (3) How much can you open up to them if you need to talk about your worries? Relationship strain was measured with the following four items: (1) How often do they make too many demands on you? (2) How much do they criticize you? (3) How much do they let you down when you are counting on them? (4) How much do they get on your nerves? Response categories were not at all = 1, a little = 2, some = 3, and a lot = 4. Responses to items from each scale were averaged to create a total score, with higher scores indicating more social support or strain. The alpha coefficients for reliability of the social support scales were  .81 for spouse/partner,  .83 for children,  .86 for family, and  .84 for friends. The reliability coefficients of the relationship strain scales were  .79 for spouse/partner,  .79 for children,  .79 for family, and  .76 for friends.

#### 2.2.2. Loneliness

We used a three-item scale that was developed to assess loneliness in large-scale surveys and that has been shown to have discriminant and convergent validity and to be related to objective measures of social isolation [25]. Respondents were asked about the frequency with which they felt they lacked companionship, felt left out, and felt isolated. Response options were often = 1, some of the time = 2, and hardly ever or never = 3. Items were reverse scored to make all items measure more frequent feelings of loneliness. The items were then averaged to create a total score ranging from 1 to 3, with higher scores indicating more feelings of loneliness. The alpha coefficient of reliability for the scale is  .81.

#### 2.2.3. Life Satisfaction

We used Diener's 5-item measure of life satisfaction, an established measure of subjective well-being [26, 27]. Respondents were asked how much they agreed or disagreed with the following five statements: (1) in most ways my life is close to ideal; (2) the conditions of my life are excellent; (3) I am satisfied with my life; (4) I have gotten the important things I want in life; and (5) if I could live my life again, I would change almost nothing. Response options were strongly disagree = 1, somewhat disagree = 2, slightly disagree = 3, slightly agree = 4, somewhat agree = 5, and strongly agree = 6. The items were averaged to create a total score ranging from 1 to 6, with higher scores indicating greater life satisfaction. The alpha coefficient for the reliability of the scale is  .87.

#### 2.2.4. Perception of Aging Experience

We measured attitudes toward the aging experience with eight items, five of which are based on the “Attitude Toward Own Aging” subscale of the Philadelphia Geriatric Center Morale Scale [28]. Questions about perceptions of the aging experience were only asked in the 2008 survey. Respondents were asked how much they agreed or disagreed with the following eight statements: (1) things keep getting worse as I get older; (2) I have as much pep as I did last year; (3) the older I get, the more useless I feel; (4) I am as happy now as I was when I was younger; (5) as I get older, things are better than I thought they would be; (6) so far, I am satisfied with the way I am aging; (7) the older I get, the more I have had to stop doing things that I like; and (8) getting older has brought with it many things that I do not like. Response options were strongly disagree = 1, somewhat disagree = 2, slightly disagree = 3, strongly agree = 4, somewhat agree = 5, and slightly agree = 6. The first, third, seventh, and eighth items were reverse scored to make all items measure positive perceptions of aging. The items were then averaged to create a total score ranging from 1 to 6, with higher scores indicating more positive perceptions of aging. The alpha coefficient for the reliability of the scale is  .82.

### 2.3. Statistical Analyses

We first examined differences in sociodemographic characteristics, living arrangements, and health status by age group. We then examined differences in social relationships between those aged 70–79 and those aged 90–104. We also examined age group differences in loneliness, life satisfaction, and perceptions of aging and show differences in both the scale items and scale means. We conducted tests of differences using the Wald chi square statistic for categorical variables and *t* tests from bivariate ordinary least squares (OLS) regression for interval variables (e.g., scale scores). Finally, we used OLS regression to determine if social relationships, health status, and psychosocial factors accounted for age group differences in loneliness, life satisfaction, and perceptions of the aging experience.

Analyses were performed using Stata software version 11 [29]. Due to the complex survey design of the HRS, we used Stata's survey prefix commands (SVY), which fit statistical models that account for the complex survey design of the HRS. All analyses, therefore, are adjusted to account for household sampling and are weighted using baseline sample weights that correct for differential probability of household selection and nonresponse and that make adjustment to the 1990 sex and age distribution of the U.S.

## 3. Results

### 3.1. Sample Characteristics

The demographic characteristics and health status of the sample by age group are presented in Table 1. Women made up the majority of the sample with more women represented among the oldest-old. The sample was mostly white and the overall racial/ethnic distribution was similar for both age groups. Among adults aged 70–79, only 22% had less than a high school education, compared to 33% of adults aged 90–104. About 61% of adults aged 70–79 were married at the time of the survey, while nearly 80% of those aged 90–104 were widowed. The high rate of widowhood among the oldest-old partly accounts for their higher rates of living alone. Both age groups had similar numbers of comorbidities, but the oldest-old had more ADL limitations. 

### 3.2. Social Relationships

Table 2 shows mean values for measures of social relationships by age group. Mean scores on the frequency of social contact measures indicate that respondents had contact with children who did not live with them on a weekly basis and had contact with other family members with whom they did not live about once or twice a month, with the oldest-old reporting more contact with children and family. Both age groups reported being in contact with friends about once or twice a month. The oldest-old reported having fewer close relationships with children but more close relationships with family members. Both age groups reported having about four close friends. 

In general, respondents reported receiving social support some of the time or a lot of the time from spouses/partners, children, family members, and friends. Those aged 90+ reported getting less social support from spouses and partners compared to the younger age group. However, compared to those aged 70–79, the oldest-old characterized their relationships with their children and other family members as having more social support and less strain. Those aged 70–79 reported having more supportive relationships with friends compared to the oldest-old.

### 3.3. Loneliness

In Table 3, we examined age differences in subjective assessments of one's sense of loneliness. About 17% of adults aged 90+ reported often feeling they lacked companionship, compared to only 10% of adults aged 70–79. The oldest-old also more often felt they were isolated compared to their younger counterparts. Age-related differences in these markers of loneliness resulted in higher scores on the loneliness scale among those aged 90+.

### 3.4. Life Satisfaction

Table 4 shows age differences in life satisfaction. We combined the somewhat and slightly agree categories and the somewhat and slightly disagree categories to create a more parsimonious comparison of item responses. There were no age differences in the amount of agreement with any of the statements about life satisfaction, except the statement that life is close to ideal. About 21% of those aged 70–79 strongly agreed with this statement compared to 17% of those aged 90+. Both age groups reported similar levels of overall life satisfaction.

### 3.5. Aging Experience

We also examined age differences in overall perceptions of and satisfaction with the aging experience.  Table 5 shows the age-specific distributions of agreement/disagreement to statements about the aging experience. We again combined the somewhat and slightly agree categories and the somewhat and slightly disagree categories to create a more parsimonious comparison of item responses. Compared to those aged 70–79, fewer oldest-old adults agreed with the positive statements about the aging experience, but more of the oldest-old agreed with negative statements about the aging experience. For instance, 19% of those aged 90+ strongly agreed with the statement “The older I get the more useless I feel” compared to only 6% of adults aged 70–79. In addition, 40% of the oldest-old strongly agreed that they have had to stop doing the things they like to do as they got older, while only 17% of those aged 70–79 felt this way. However, mean differences in the aging experience scale indicate that in general, the oldest-old adults had a less positive overall perception of their aging experience.

### 3.6. Multivariate Analysis

Table 6 presents coefficients from the OLS regression models for loneliness (Panel A), life satisfaction (Panel B), and perceptions of the aging experience (Panel C). Panel A shows the results for loneliness. The first model includes dichotomous indicators for age group, gender and race/ethnicity, and a continuous measure of years of education. Loneliness was higher among the oldest-old and women and declined with increasing education. 

The second model adds marital and living status as indicators of social contact. Although we also examined other indicators of social contact, marital status had consistently stronger and more significant associations with the outcome measures (results not shown). Not being married was associated with more frequent feelings of loneliness, even with an adjustment for living alone. After accounting for differences in marital status, the age difference in loneliness was reduced and no longer statistically significant. Nearly 80% of the oldest old were widowed, which seems to explain why the oldest-old report more frequent feelings of loneliness compared to those aged 70–79. This explanation seems particularly plausible considering that one of the items in the loneliness scale asks about lack of companionship which tends to be provided by a spouse or partner. 

After adjusting for comorbidities and ADL limitations in the next model, the coefficient for the age difference became negative and was marginally significant (−.07, *P* < .10). The number of comorbidities and ADL limitations was associated with greater feelings of loneliness. The number of ADL limitations increases with age, suggesting that feelings of loneliness may be less frequent among the oldest-old who have similar ADL profiles as those aged 70–79. 

Panel B presents the results for the OLS regression model for life satisfaction. The first column shows that life satisfaction was lower among Blacks and that satisfaction increased with increasing education. However, there were no age differences in life satisfaction. 

The second model adds social contact indicators and shows that not being married was associated with less life satisfaction. Moreover, after adjusting for social contact, the coefficient for the age differences increased in magnitude and became statistically significant (.19, *P* < .05). This suggests that the oldest-old may have greater life satisfaction than younger elderly adults when differences in marriage and widowhood are accounted for. The age difference increased further in the next model, which adjusts for differences in comorbidities and ADL limitations, and shows that greater levels of disease and functional limitation were associated with less life satisfaction. Taken together, these two models suggest that differences in life satisfaction between the old and oldest-old are underestimated when age-related differences in marital and health status are not considered. 

In the final model, we also adjusted for loneliness. Greater feelings of loneliness were associated with less life satisfaction. The coefficients for comorbidities and ADL limitations were reduced after including loneliness, which indicates that the negative associations between health status and life satisfaction may operate partially through increased feelings of loneliness among those who experience poor health and activity limitations.

Panel C shows the results for perceptions of the aging experience. The first column shows that oldest-old adults had more negative perceptions of their aging experience and that having more years of education was associated with more positive perceptions of the aging experience. The second column shows that those who were not married had more negative perceptions of aging, but that accounting for differences in marital status did not explain the age difference. 

The third model also included comorbidities and ADL limitations and shows that individuals who had worse physical health and functioning reported more negative perceptions of their aging experience. After accounting for differences in health status, the coefficient for the age difference was reduced by about 50%. The final model shows that feelings of loneliness were associated with more negative perceptions of aging, but that loneliness did not account for age differences in perceptions.

## 4. Discussion

The purpose of this study was to gain insight into the social and psychological well-being of the oldest-old US adults, using a younger group of older adults as a comparison. We add to previous research that has used traditional biomedical-based models for understanding aging and longevity by examining psychosocial factors associated with longevity. 

We found that the oldest-old had frequent social contact with family and friends and relatively high levels of social support. The high amount of social contact reported by the oldest-old in our study is consistent with other samples of oldest-old from the US and England [30, 31]. However, our finding that the oldest-old report having more contact with children and family than younger elderly adults is contrary to what has been reported in a prior study of US oldest-old adults [32]. Differences between our results and results from previous research may be due to differences in the measure but may also be due to differences in the study sample; ours was a national study of US adults aged 90 and older and the previous study focused on centenarians living in Georgia. 

Compared to younger elderly adults, the oldest-old in our study reported receiving more positive support from their children and family. This finding is consistent with previous research [24]. The oldest-old also reported less strained relationships with their children. Overall, our results indicate that social relationships remain intact in the oldest-old. Research shows that social relationships are generally beneficial for health and well-being and thus, maintaining relationships with family and friends may be a key component of quality of life in advanced old age [16].

We also found evidence that perceptions of quality of life are lower among the oldest-old. Consistent with prior work [23], we found that the oldest-old felt lonely more often than younger elderly adults. By looking at multiple indicators of loneliness, we additionally found that age differences in loneliness are particularly pronounced with respect to lacking companionship and feeling isolated. However, the results indicate that the oldest-old have greater feelings of loneliness, because they do not have a spouse or partner to provide companionship, and because they have health limitations that may limit their social contact. 

In accordance with prior research, we found very old adults were more satisfied with life than younger elderly adults [24] but only after accounting for marital status, comorbidities and ADL limitations, and loneliness. The age difference in life satisfaction was underestimated when social and health factors were not considered. This suggests that the social and health conditions that typically accompany old age, for instance losing a spouse and having difficulty with daily activities, decrease life satisfaction among the oldest-old, but that in the absence of these factors, long-lived individuals are more satisfied with their lives than younger elderly adults.

We also found that the oldest-old had more negative perceptions of their aging experience. For instance, 69% of the oldest-old agreed that things had gotten worse as they aged, 53% agreed that they felt more useless as they got older, and 88% agreed that they had to stop doing things they like to do. The higher burden of activity limitations among the oldest-old partially accounted for their more negative perceptions of the aging experience. Declines in physical functioning can prevent older adults from doing the things they want to do and may be a primary reason they feel useless and that their lives have gotten worse. 

Even though the oldest-old were less satisfied with the aging experience, they reported greater life satisfaction. This may reflect the tendency of the oldest-old to reconstitute how they view themselves and their experiences to be more consistent with the realities of their lives [33]. So, even though the oldest-old express dissatisfaction with particular aspects of the aging experience, they may still believe that their overall life situation is as good as that of younger elderly adults. 

Taken together, the results show that the oldest-old have similar, if not more, social contact than younger old adults but that the oldest-old still feel more lonely and socially disconnected, which is most likely to due to decreased social contact and interaction as a result of their higher rates of widowhood, disease, and disability. The results also show that the oldest-old have greater overall life satisfaction but more negative perceptions of the aging experience. 

This study is the first to examine social and psychological well-being associated with aging and longevity in a national sample of US adults. We use several measures of psychosocial well-being to provide a comprehensive understanding of quality of life at advanced old age. The findings from this study make important contributions to the growing body of research on longevity and quality of life. In particular, this study highlights the importance of considering psychosocial factors associated with longevity in addition to other social and health factors that characterize the aging experience.

This study has some limitations. First, although we used a representative sample of US adults, information on psychosocial characteristics was not obtained from older adults living in nursing homes. Nursing home residents are more likely to have functional and cognitive impairments, and this sample may, therefore, have better health and functioning than the general population of adults aged 70–79 and especially adults aged 90 and older. Thus, the study results cannot be generalized beyond the community-dwelling older adult population as it is possible that quality of life in long-lived nursing home residents differs from that of community-dwelling oldest-old [22]. 

Another limitation is that the age differences reported in this study may be confounded with cohort differences. Although our results may indicate that psychosocial factors change with age, it is also possible that the age differences found in our study additionally or, instead, reflect cohort differences in how psychosocial factors relate to age.

## 5. Conclusions

Our findings suggest that the oldest-old are able to maintain quality of life with respect to social relationships and that while their aging experience to that point has been difficult, they are as satisfied with their lives as younger elderly adults. Our study contributes to the growing research on quality of life among the very old.

A consideration of psychosocial factors associated with longevity is essential not only for predicting longevity, but also for understanding quality of life among the longest lived individuals. Complex associations exist between health and functioning and psychosocial well-being [34], and the joint influence of these factors on longevity and quality of life should be considered in future longevity research [35].

## Figures and Tables

**Table 1:** Sample characteristics by age group.

	Age 70–79	Age 90–104	*P* value
	*N *(%)	*N *(%)	
Female	2,374 (56.3)	200 (69.6)	<.000
Male	1,813 (43.7)	81 (30.4)	
Race/ethnicity			
White	3,332 (85.3)	230 (86.5)	n.s.
Black	510 (7.9)	29 (6.8)	
Hispanic	272 (4.9)	19 (5.9)	
Other	73 (1.9)	3 (0.8)	
Completed education			
Less than high school	929 (21.9)	92 (32.6)	<.000
High school	1,662 (39.4)	96 (33.6)	
College or more	1,593 (38.7)	93 (33.8)	
Marital Status			
Married/partnered	2,698 (61.1)	49 (16.5)	<.000
Divorced/separated	387 (10.2)	7 (2.6)	
Widowed	994 (25.7)	222 (79.9)	
Never married	108 (2.9)	3 (0.9)	
Lives alone	1,053 (27.0)	170 (59.1)	<.000
Comorbidities			n.s.
0	680 (16.7)	36 (11.5)	
1	1,400 (33.2)	94 (32.4)	
2	1,211 (28.6)	95 (33.1)	
3	623 (14.7)	41 (17.4)	
4+	273 (6.9)	15 (5.7)	
ADL limitations			<.000
0	3,478 (81.9)	149 (49.7)	
1	364 (8.9)	58 (21.1)	
2	160 (4.0)	32 (11.3)	
3	88 (2.3)	20 (7.9)	
4+	97 (2.9)	22 (10.0)	

Total *N *	*N* = 4,187	*N* = 281	

Note: Figures shown are weighted sample sizes with percentages in parentheses. *P* values denoting statistical significance of age differences were obtained using Wald chi square tests.

ADLs: activities of daily limitations.

**Table 2:** Age differences in social relationships between the old (age 70–79) and the oldest-old (age 90–104), HRS 2006/2008.

	Age 70–79			Age 90–104			*P*
	N	Mean	(s.d.)	N	Mean	(s.d.)	
Social contact							
Children	1933	4.54	(1.28)	108	4.93	(1.20)	<.001
Family	1931	3.80	(1.45)	114	4.10	(1.60)	.048
Friends	1939	4.36	(1.25)	113	4.38	(1.19)	n.s.
Close relationships							
Children	3854	2.85	(2.11)	226	2.45	(2.22)	.009
Family	3789	3.43	(3.17)	226	3.98	(3.50)	.020
Friends	3761	3.79	(3.29)	233	4.07	(3.71)	n.s.
Relationship quality							
Spouse/partner							
Social support	2710	3.50	(0.70)	65	3.31	(0.75)	.048
Strain	2735	1.95	(0.77)	69	1.82	(0.82)	n.s.
Children							
Social support	3813	3.35	(0.77)	225	3.54	(0.66)	<.001
Strain	3840	1.61	(0.66)	230	1.50	(0.68)	.025
Family							
Social support	3834	2.91	(0.98)	225	3.10	(0.88)	<.001
Strain	3847	1.48	(0.62)	228	1.36	(0.54)	<.001
Friends							
Social support	3894	3.03	(0.85)	238	2.95	(0.89)	n.s.
Strain	3819	1.37	(0.50)	243	1.31	(0.43)	.031

Note: Figures shown are weighted sample sizes and means with standard deviation in parentheses. *P* values denoting statistical significance of age differences were obtained using ANOVA *F* tests.

Social contact with children, family, and friends was measured in 2008 only.

**Table 3:** Differences in loneliness between the old (age 70–79) and the oldest-old (age 90–104), HRS 2006/2008.

	*N*	Often	Sometimes	Hardly ever/Never	*P*
Lack companionship					
Age 70–79	4,109	9.9	34.0	56.1	<.000
Age 90–104	269	17.1	39.7	43.2	
Feel left out					
Age 70–79	4,095	6.1	33.3	60.6	n.s.
Age 90–104	265	7.4	37.4	55.2	
Feel isolated					
Age 70–79	4,082	6.3	26.1	67.7	.085
Age 90–104	265	10.3	26.8	62.9	

Loneliness scale		Mean (s.d.)			
Age 70–79	4,123	1.46 (0.61)			<.001
Age 90–104	274	1.58 (0.61)			

Note: Figures shown are weighted sample sizes and percentages and weighted scale means with standard deviation in parentheses. *P* values denoting statistical significance of age differences were obtained using Wald chi square tests for the items and *t* tests from bivariate OLS regression for the scale mean.

**Table 4:** Differences in life satisfaction between the old (age 70–79) and the oldest-old (age 90–104), HRS 2006/2008.

	*N*	Strongly agree	Somewhat/ slightly agree	Somewhat/ slightly disagree	Strongly disagree	*P*
Life is close to ideal						
Age 70–79	3,780	20.9	55.4	17.0	6.7	.063
Age 90–104	240	16.8	56.0	20.6	6.6	
Conditions of life are excellent						
Age 70–79	3,866	20.9	52.5	19.3	7.3	n.s.
Age 90–104	243	20.4	52.6	19.6	7.4	
Satisfied with life						
Age 70–79	3,974	36.3	47.4	11.7	4.6	n.s.
Age 90–104	253	34.6	47.3	13.0	5.2	
Gotten important things in life						
Age 70–79	3,967	33.1	52.5	10.9	3.6	n.s.
Age 90–104	260	35.5	51.0	9.4	4.2	
Would not change life						
Age 70–79	3,926	19.5	46.3	22.5	11.6	n.s.
Age 90–104	251	25.9	42.4	21.4	10.3	

Life satisfaction scale		Mean (s.d.)				
Age 70–79	4,095	4.40 (1.45)				n.s.
Age 90–104	269	4.39 (1.27)				

Note: Figures shown are weighted sample sizes and percentages and weighted scale means with standard deviation in parentheses. *P* values denoting statistical significance of age differences were obtained using Wald chi square tests for the items and *t* tests from bivariate OLS regression for the scale mean.

**Table 5:** Differences in perceptions of the aging experience between the young old (age 70–79) and the very old (age 90–104), HRS 2008.

	*N*	Strongly agree	Somewhat/ slightly agree	Somewhat/ slightly disagree	Strongly disagree	*P*
Things get worse as I get older						
Age 70–79	2,054	9.3	49.3	24.7	16.6	.019
Age 90–104	135	12.1	56.6	21.4	9.9	
I have as much pep as last year						
Age 70–79	2,061	16.2	39.1	34.9	9.7	<.000
Age 90–104	137	10.7	29.4	40.8	19.2	
The older I get the more useless I feel						
Age 70–79	2,050	5.9	25.8	29.9	38.4	<.000
Age 90–104	134	18.5	34.5	28.1	18.9	
I am as happy now as I was when I was younger						
Age 70–79	2,062	24.8	36.9	26.0	12.3	<.000
Age 90–104	135	10.2	34.2	38.4	17.2	
As I get older things are better than I thought they would be						
Age 70–79	2,057	22.2	48.3	22.4	7.2	n.s.
Age 90–104	135	19.4	44.2	23.1	13.4	
I am satisfied with the way I am aging						
Age 70–79	2,068	31.2	49.4	13.7	5.8	n.s.
Age 90–104	137	38.2	45.0	11.2	5.6	
The older I get, the more I have had to stop doing things that I liked						
Age 70–79	2,069	17.2	49.0	21.7	12.2	<.000
Age 90–104	135	40.4	47.4	9.1	3.1	
Getting older has brought with it many things that I do not like						
Age 70–79	2,069	17.3	52.4	19.9	10.5	<.000
Age 90–104	136	31.2	54.7	7.9	6.2	

Aging experience scale		Mean (s.d.)				
Age 70–79	2,073	3.81 (1.25)				<.000
Age 90–104	137	3.30 (0.94)				

Note: Figures shown are weighted sample sizes and percentages and weighted scale means with standard deviation in parentheses. *P* values denoting statistical significance of age differences were obtained using Wald chi square tests for the items and *t* tests from bivariate OLS regression for the scale mean.

Perceptions of the aging experience were measured in 2008 only.

**Table 6:** OLS regression models for loneliness, life satisfaction, and perceptions of the aging experience.

	Base model	+ Social contact	+ ADLs	+ Loneliness
	Coeff. (SE)	Coeff. (SE)	Coeff. (SE)	Coeff. (SE)
*Panel A. Model for Loneliness *				

Age 90–104	.09 (.04)*	−.02 (.04)	−.07 (.04)^+^	
Female	.09 (.02)***	.01 (.02)	.01 (.02)	
Black^a^	.06 (.03)^+^	.01 (.03)	−.01 (.03)	
Hispanic^a^	.05 (.05)	.04 (.05)	.04 (.05)	
Other^b^	−.07 (.06)	−.07 (.06)	−.06 (.06)	
Education, yrs	−.02 (.00)***	−.02 (.00)***	−.01 (.00)***	
Not married		.24 (.03)***	.22 (.03)***	
Lives alone		.02 (.03)	.04 (.03)	
Comorbidities			.03 (.01)***	
1 ADL limitation^b^			.15 (.03)***	
2+ ADL limitations^b^			.20 (.03)***	
Constant	1.68 (.05)***	1.59 (.04)***	1.46 (.05)***	
*R* squared	0.030	0.075	0.100	

*Panel B. Model for Life Satisfaction *				

Age 90–104	.03 (.08)	.19 (.08)*	.33 (.09)***	.26 (.08)***
Female	−.05 (.04)	.08 (.04)*	.06 (.04)	.06 (.04)^+^
Black^a^	−.23 (.07)***	−.17 (.07)*	−.11 (.07)	−.11 (.07)
Hispanic^a^	.16 (.10)	.17 (.10)^+^	.16 (.10)	.13 (.09)
Other^b^	.34 (.12)**	.34 (.12)**	.33 (.11)**	.26 (.11)*
Education, yrs	.04 (.01)***	.04 (.01)***	.03 (.01)***	.02 (.01)*
Not married		−.38 (.07)***	−.30 (.07)***	−.12 (.07)^+^
Lives alone		−.04 (.08)	−.10 (.07)	−.07 (.07)
Comorbidities			−.12 (.02)***	−.09 (.02)***
1 ADL limitation^b^			−.39 (.07)***	−.28 (.07)***
2+ ADL limitations^b^			−.52 (.08)***	−.38 (.08)***
Loneliness				−.79 (.04)***
Constant	3.88 (.11)***	4.03 (.11)***	4.45 (.11)***	5.61 (.12)***
*R* squared	0.017	0.040	0.081	0.193

*Panel C. Model for Aging Experience *				

Age 90–104	−.47 (.09)***	−.42 (.09)***	−.21 (.09)*	−.24 (.08)**
Female	.02 (.05)	.06 (.05)	.04 (.05)	.02 (.04)
Black^a^	.04 (.08)	.08 (.09)	.16 (.07)*	.15 (.07)*
Hispanic^a^	.13 (.11)	.14 (.11)	.09 (.11)	.16 (.10)
Other^b^	−.11 (.18)	−.11 (.18)	−.11 (.18)	−.13 (.17)
Education, yrs	.06 (.01)***	.06 (.01)***	.04 (.01)***	.03 (.01)***
Not married		−.18 (.08)*	−.01 (.08)	.12 (.07)
Lives alone		.08 (.08)	−.06 (.08)	−.04 (.07)
Comorbidities			−.17 (.02)***	−.49 (.08)***
1 ADL limitation^b^			−.62 (.08)***	−.66 (.08)***
2+ ADL limitations^b^			−.84 (.08)***	−.16 (.02)***
Loneliness				−.68 (.04)***
Constant	3.05 (.13)***	3.10 (.13)***	3.68 (.13)***	4.73 (.14)***
R squared	0.042	0.045	0.175	0.282

Notes: Numbers are coefficients with standard errors in parentheses.

^a^Reference group is white; ^b^Reference group is no ADLs.

***P < .001, **P < .01, *P < .05, ^+^ *P* < .10 (two-tailed test).
